# Regenerative peripheral nerve interface following forequarter amputation: a case report

**DOI:** 10.3389/fsurg.2026.1771682

**Published:** 2026-04-30

**Authors:** Jinxian Zhao, Weichun Liang, Mingshuang Wang, Haiwen Pan, Jianfeng Chen, Qingbin Li, Jianhui Lin, Guokai Feng, Zongquan Mo, Yongqiang Lao

**Affiliations:** 1 Foshan Hospital of Traditional Chinese Medicine, The Eighth Clinical Medical College, Guangzhou University of Chinese Medicine, Guangzhou, China; 2 Department of Osteopathy, Foshan Hospital of Traditional Chinese Medicine, Foshan, Guangdong Province, China

**Keywords:** forequarter amputation, neuropathic pain, osteosarcoma, phantom limb pain, regenerative peripheral nerve interface

## Abstract

**Background:**

Forequarter amputation is a radical surgical procedure for malignant bone and soft tissue tumors involving the shoulder girdle, typically indicated for cases where limb salvage is not feasible due to extensive local invasion. However, post-amputation neuroma and phantom limb pain are major complications, occurring in 80%–90% of cases and severely impairing patients' quality of life. Traditional nerve management techniques often fail to prevent recurrent neuroma formation. The Regenerative Peripheral Nerve Interface (RPNI) is an emerging technique designed to guide axonal regeneration and mitigate pain. However, its application in oncologic high-level amputations has not been widely reported.

**Methods:**

We report the case of a 68-year-old female who underwent forequarter amputation for a malignant tumor of the left upper arm with simultaneous prophylactic RPNI reconstruction. The main trunks of the brachial plexus were sharply transected, and their stumps were implanted into small segments of denervated free muscle graftsharvested from the brachioradialis muscle of the amputated limb to promote organized axonal regeneration.

**Results:**

The patient's postoperative course was uneventful, without infection or other complications. Follow-ups at 1, 3, 6, and 12 months demonstrated significant and sustained relief of residual limb pain. The Visual Analog Scale (VAS) score decreased from a preoperative level of 9 to 0 Crucially, the patient reported no phantom limb pain or symptomatic neuroma-related discomfort during follow-up. Final pathology confirmed osteosarcoma.

**Conclusion:**

Concurrent RPNI reconstruction during forequarter amputation for malignancy is a safe and effective strategy to prevent postoperative neuropathic pain. This innovative application in high-level amputation patients offers a promising approach to improving quality of life.

## 1 Introduction

Malignant tumors of the shoulder girdle often involve the glenohumeral joint, proximal humerus, and surrounding musculature (1). For cases with extensive local invasion where limb salvage is not feasible, forequarter amputation remains a necessary procedure to achieve oncological control (2). However, this high-level, extensive amputation is associated with severe neuropathic pain and phantom limb pain, reported in over 80% of cases, significantly hindering rehabilitation and impairing quality of life (3). Traditional nerve management techniques, such as neurotomy or burying nerve ends in adjacent muscle or periosteum (4), are often insufficient in preventing the disorganized axonal regeneration that underlies neuropathic pain.

In recent years, the Regenerative Peripheral Nerve Interface (RPNI) technique has emerged as a novel approach to address post-amputation pain challenges (5). First described by Dijkstra et al. (6) the fundamental principle of RPNI involves implanting the transected nerve stump into a small, free denervated muscle graft. This muscle graft acts as a biological target, providing metabolic support and an organized microenvironment for regenerating axons, thereby preventing neuroma formation and alleviating neuropathic pain.

Current literature on RPNI primarily focuses on traumatic amputations. To our knowledge, reports on its concurrent applicationin oncological high-level amputations remain scarce. This case report details such a complex procedure, aiming to explore the technical feasibility and clinical efficacy of RPNI in tumor-related forequarter amputation.

## 2 Case report

### 2.1 Patient history

A 68-year-old female was admitted in November 2024 for a massive malignant sarcoma of the left upper arm. Preoperative MRI revealed extensive tumor involvement of the left deltoid, teres minor, teres major, and triceps brachii muscles. Despite completing three cycles of neoadjuvant chemotherapy combined with anlotinib targeted therapy, the tumor volume remained substantial. To achieve radical resection, a left forequarter amputation with concurrent RPNI reconstruction was planned.

The patient's symptoms began eight months prior, presenting as swelling and pain in the left upper arm, limited left shoulder mobility, and numbness distal to the left elbow. A previous incisional biopsy at an outside hospital suggested a mesenchymal malignant tumor, consistent with an undifferentiated pleomorphic sarcoma with abundant giant cells. From December 2024 to April 2025, she completed an additional 6 cycles of neoadjuvant chemotherapy combined with anlotinib targeted therapy.

### 2.2 Preoperative examination

Significant swelling was noted in the left upper extremity, most pronounced in the upper arm and shoulder. The maximum circumference of the left upper arm was 47 cm, compared with 31 cm on the contralateral (right) side; The left shoulder circumference was 58 cm, compared with 40 cm on the contralateral (right) side. Extensive skin pigmentation was observed over the left upper arm. A large soft tissue mass was palpable in the left upper arm and shoulder. Left shoulder and elbow range of motion was limited. Distal circulation was adequate with a palpable radial pulse. A left wrist drop with limited dorsiflexion was noted. Numbness was noted below the left elbow. Thumb abduction and extension of digits 2–4 were slightly limited.

### 2.3 Surgical procedure

Following preoperative evaluation, the patient underwent left forequarter amputation under general anesthesia on November 5, 2024. Positioned in the right lateral decubitus position, a curved incision was made from the medial third of the clavicle, over the acromion, along the scapular border to the posterior axilla. Muscles including the trapezius, levator scapulae, rhomboids, serratus posterior, and latissimus dorsi were divided. An anterior incision along the deltopectoral groove allowed division of the deltoid, pectoralis major, pectoralis minor, and omohyoid muscles. The clavicle was osteotomized. The subclavian vessels were identified, doubly ligated, and transected.

RPNI Reconstruction: After fully exposing the major trunks of the brachial plexus and infiltrating them with local anesthetic for block, the nerves were sharply transected, and their stumps were freed. RPNI reconstruction was performed using free denervated muscle grafts: a segment of the brachioradialis muscle was harvested from the amputated specimen as the free muscle graft, measuring 30 × 15 × 15 mm. The graft was harvested along the direction of the brachioradialis muscle fibers, with excess fibrous connective tissue removed. A small portion of the original vascular pedicle was intentionally preserved to facilitate rapid initial integration with the recipient site.

**Figure 1:**
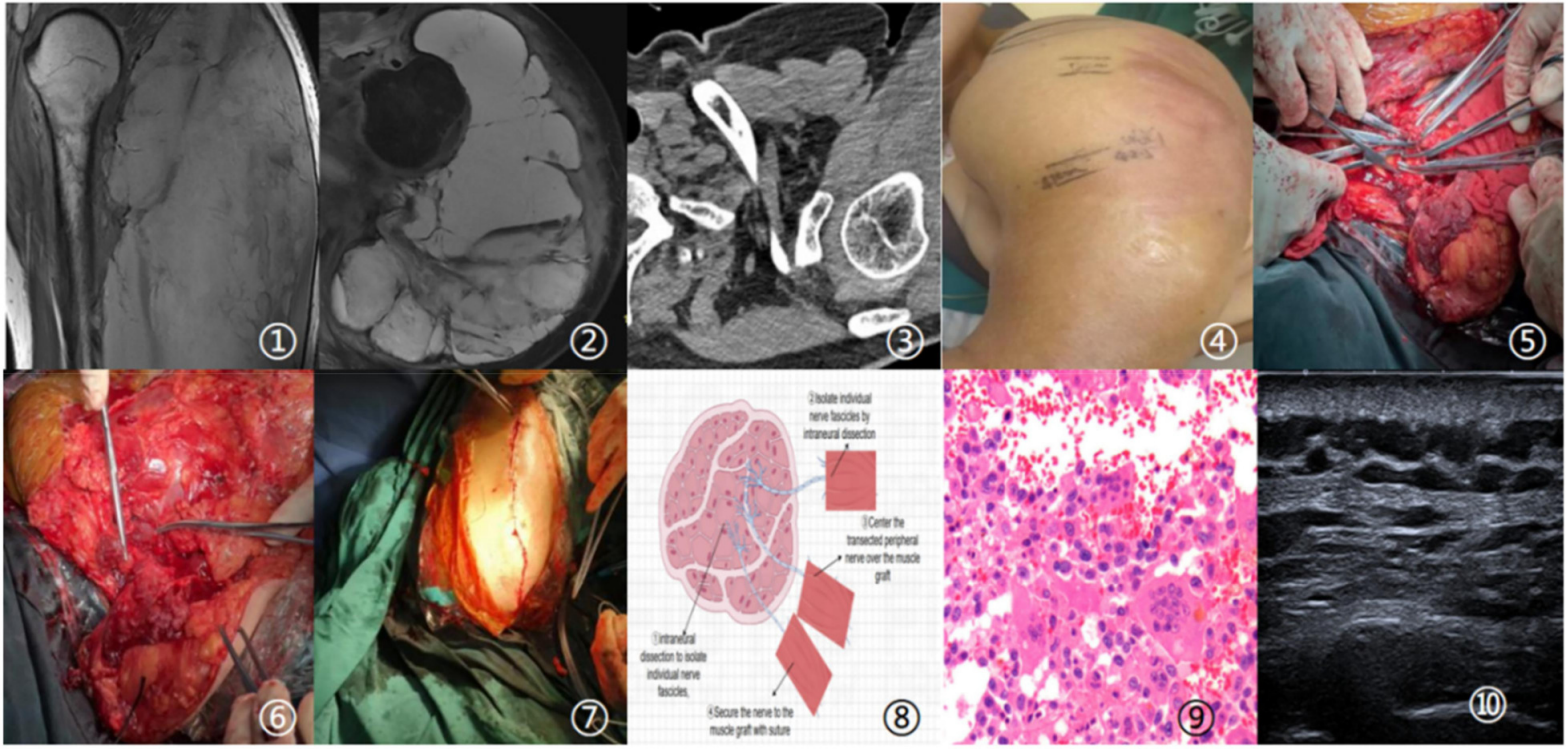
① and ②: Preoperative MRI images showing tumor invasion of the muscular tissues; ③:preoperative CT scan; ④:preoperative measurement of the maximum arm circumference (47 cm); ⑤: intraoperative exposure of the brachial plexus tissue; ⑥: wrapping of the brachial plexus stumps with muscle tissue; ⑦: sutured surgical wound; ⑧: schematic diagram of the regenerative peripheral nerve interface (RPNI) procedure; ⑨: postoperative pathological findings; ⑩: postoperative 1-year follow-up ultrasound image.

The major stumps of the brachial plexus were then freed and individually implanted into these normal muscle tissues to construct the RPNI, guiding ordered axonal regeneration. Three muscle grafts were implanted during the operation: the stump of the upper trunk of the brachial plexus was implanted into the first graft, the stump of the middle trunk into the second graft, and the stump of the lower trunk into the third graft. Due to shortening of the lower trunk after injury, peritumoral tissue release was performed as shown in Figure 1 (Item ⑤). Before transection, the lower trunk was clamped with curved forceps by multiple surgeons to prevent shortening and displacement.

The surgical field was thoroughly hemostasized and irrigated. Two closed negative-pressure drainage tubes were placed from the lower end of the incision. The skin flap was trimmed, and the wound was sutured layer by layer. Local injection of tranexamic acid was administered to achieve hemostasis. The amputated specimen was sent for pathological examination after the operation.

## 3 Results

Intraoperative blood loss was 1500 mL. The patient recovered well without infection or other complications. Drains were removed on postoperative day 4, and the patient was discharged on day 10.

Pain Assessment: At the one-year follow-up, the patient reported significant relief of residual limb pain. The VAS score decreased from a preoperative 9 to 0. Notably, the patient denied phantom limb pain or any discomfort suggestive of neuroma formation.

**Figure 2:**
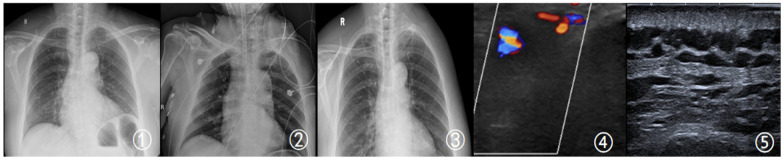
①: Preoperative 3-month DR image of the patient; ②: postoperative follow-up DR image; ③: postoperative 3-month follow-up DR image; ④: preoperative color Doppler ultrasound image, with no vascular abnormalities detected; ⑤: postoperative 1-year follow-up color Doppler ultrasound image of the patient: patent blood vessels, with no abnormalities detected.

**Figure 3:**
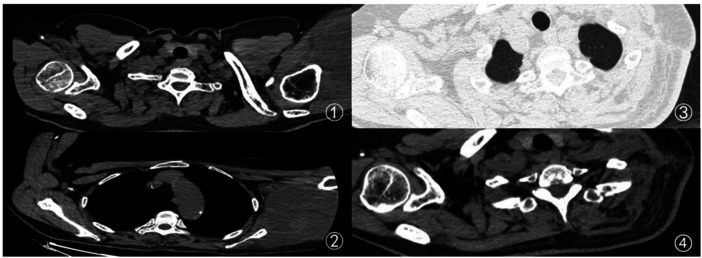
①: Preoperative 3-month CT scan of the patient, showing swelling of the left upper arm; ②: preoperative CT scan of the patient after completing 3 cycles of chemotherapy; ③: postoperative 6-month follow-up CT scan, with no tumor recurrence detected; ④: postoperative 1-year follow-up CT scan of the patient, with no tumor recurrence detected.

**Figure 4:**
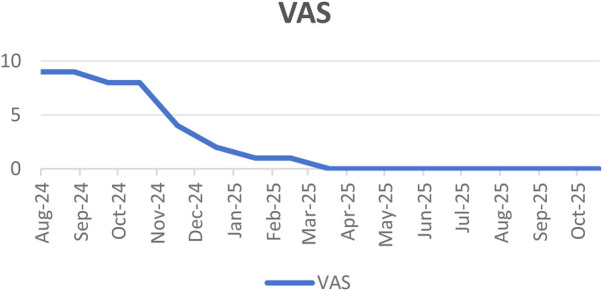
Dynamic changes in the patient's VAS scores during 1-year follow-up.

Pathology: Final pathological diagnosis was well-differentiated osteosarcoma, with approximately 80% tumor necrosis and 20% residual viable tumor, confirming the efficacy of neoadjuvant therapy (Figures 2–4).

## 4 Discussion

While forequarter amputation achieves local tumor control, the high incidence of subsequent chronic neuropathic pain remains a significant clinical challenge. Traditional nerve management techniques, such as nerve burial, have demonstrated limitations due to suboptimal long-term efficacy and a high rate of neuroma recurrence (7).

The Regenerative Peripheral Nerve Interface (RPNI) has been shown to effectively alleviate phantom limb pain, reduce the incidence of neuroma (8), and mitigate post-amputation pain (9). Its core mechanism entails implanting transected nerve stumps into free denervated muscle grafts. This guides axonal growth into the target muscle, thereby preventing disorganized axonal sprouting and neuroma formation while enabling targeted reinnervation (10). The free muscle graft is typically harvested from healthy muscle tissue of the amputated limb. Existing literature highlights the importance of graft size: excessively thick grafts are at risk of necrosis, whereas grafts that are overly thin or inadequately denervated may fail to support sufficient signal amplification (5). A recommended graft dimension is approximately 30 × 15 × 15 mm (SCI standard for measurement notation) (11), harvested parallel to the muscle fiber direction to minimize tissue damage and with all connective tissue meticulously trimmed to optimize axonal regeneration. In the present case (12), a 30 × 15 × 10 mm graft harvested from the brachioradialis muscle was utilized.

In contrast to Targeted Muscle Reinnervation (TMR), RPNI does not require an intact donor motor nerve, is technically less complex, and results in reduced surgical trauma. In the context of this high-level amputation, RPNI enabled the use of a free muscle graft from the amputated specimen, thereby simplifying the procedure in a clinical setting requiring single-stage radical tumor resection. This represents a key advantage over TMR, which typically relies on innervated donor muscles and may be less feasible in extensive oncological resections (13).

The novelty of the present case resides in the concurrent performance of RPNI reconstruction during a complex oncological forequarter amputation. The successful utilization of a brachioradialis muscle graft harvested from the amputated limb (i.e., local graft harvesting) eliminated the need for additional donor site dissection, thereby avoiding donor site morbidity and confirming the technical feasibility and safety of RPNI in the setting of complex tumor resections (13). A 75% reduction in VAS score was observed at the one-year follow-up. Moreover, phantom limb pain was absent in these patients. These findings strongly validate the clinical value of RPNI in patients undergoing high-level amputations. To our knowledge, this is one of the first reports of RPNI application in tumor-related forequarter amputation, expanding the utility of this nerve interface technology beyond traumatic amputations (14).

A major limitation of the present study is its single-case design, which lacks validation from large-scale multicenter randomized controlled trials (RCTs). Additionally, long-term electrophysiological data (e.g., electromyographic assessment of graft reinnervation) were not available to objectively confirm axonal integration into the RPNI graft. Future research should focus on the long-term functional evaluation of RPNI interfaces, utilizing electrophysiological and advanced imaging modalities (e.g., diffusion tensor imaging) to verify signal amplification capacity and axonal integration.

## 5 Conclusion

The application of the Regenerative Peripheral Nerve Interface (RPNI) technique during tumor-related forequarter amputation is safe, technically feasible, and effective for preventing neuroma formation and alleviating phantom limb pain. This report describes our experience with a surgical strategy for managing post-amputation pain that integrates advanced neural interface technology, aiming to enhance understanding of therapeutic strategies for amputees and provide a novel approach for the prevention and management of chronic neuropathic pain. The technique confers several advantages, including relative technical simplicity, convenient graft accessibility (utilizing the amputated limb as the graft source), and a low complication rate. Future large-scale multicenter studies are warranted to further validate the long-term efficacy of RPNI and explore its expanded applications, such as in prosthetic control and enhanced neural regeneration (15).

## Data Availability

The original contributions presented in the study are included in the article/Supplementary Material, further inquiries can be directed to the corresponding author.
